# Caregiver coping mediates the relationship between caregivers’ understanding of dementia as terminal and their distress

**DOI:** 10.1002/alz.14102

**Published:** 2024-07-06

**Authors:** Ishwarya Balasubramanian, Louisa Camille Poco, Ellie Bostwick Andres, Isha Chaudhry, Truls Østbye, Chetna Malhotra, PISCES study group

**Affiliations:** ^1^ Lien Centre for Palliative Care Duke‐NUS Medical School Singapore Singapore; ^2^ Family Medicine and Community Health Duke University Durham North Carolina USA; ^3^ Health Services and Systems Research Duke‐NUS Medical School Singapore Singapore

**Keywords:** caregiver, coping, dementia, distress, mediation, prognostic understanding, terminal illness

## Abstract

We assessed which coping strategies (problem‐focused, emotion‐focused, dysfunctional) mediate the association between caregivers’ understanding of dementia as terminal and their distress. A total of 215 caregivers of community‐dwelling persons with severe dementia were surveyed every 4 months over 3 years. A generalized structural equation model was used to test mediation. Caregivers who correctly understood dementia as terminal and those unsure (vs incorrect), experienced more distress (correct: *β* [95% confidence interval (CI)]: 0.80 [0.00 to 1.60]; unsure: 0.95 [0.04 to 1.87]). Caregivers with correct understanding of dementia as terminal (vs incorrect) employed more dysfunctional (2.01 [0.60 to 3.42]) and problem‐focused coping strategies (2.56 [0.08 to 5.05]). Although dysfunctional and problem‐focused coping (associated with higher distress) mediated the positive association between caregivers’ understanding that dementia is terminal and their distress, emotion‐focused coping (associated with lower distress) did not offset this relationship. Results suggest that terminal illness disclosure to caregivers should be accompanied by interventions to promote emotion‐focused coping strategies.

**Highlights:**

- Caregivers who understood dementia as terminal experienced more distress.
- Dysfunctional and problem‐focused coping mediated the positive relationship between terminal illness understanding and caregiver distress.
- Emotion‐focused coping did not offset this relationship.

## 1 BACKGROUND

Dementia currently affects over 55 million people worldwide and is estimated to double every 20 years.^1^ Severe dementia is characterized by severe functional deficits. Pneumonia, febrile episodes, and dysphagia are common problems at this stage, ultimately leading to death.^2^ Given that severe dementia is a terminal illness, adopting a person‐centered and palliative care approach is considered appropriate.^3^ Caregivers who recognize dementia as terminal are more likely to access palliative care and offer comfort‐focused care to persons with severe dementia (PwSDs).^4^, ^5^ However, the effect of caregivers’ understanding of PwSDs’ terminal illness on their own distress levels may vary. Some caregivers may accept the terminal nature of illness, allowing them to be more prepared for future end‐of‐life (EOL) decisions and experience reduced distress. Others may struggle to fully accept the terminal nature of PwSDs’ illness, leading to self‐blame and increased distress. Existing studies exploring the association between terminal illness understanding and distress have predominantly focused on patients with advanced cancer, yielding conflicting results.^6^, ^7^, ^8^, ^9^ However, the relationship between terminal illness understanding and distress has been relatively underexplored among caregivers, especially those of PwSDs.^10^

The common sense model developed by Leventhal and colleagues^11^ and Lazarus and Folkman's model of stress and coping^12^ posit that the impact of an illness on an individual is determined by how that individual perceives the illness, with the effects of perception being mediated by their chosen coping strategy. Applying this model in the context of caregivers of PwSDs, we posit that caregivers’ perception of dementia as a terminal illness influences their distress, with the association being mediated by how they cope with this knowledge.^13^

Carver et al.^14^ identified three types of coping strategies: problem‐focused, emotion‐focused, and dysfunctional. Problem‐focused coping involves active efforts by the caregiver to mitigate the stressor. Emotion‐focused coping entails caregivers’ efforts to modify their emotional response to the stressor rather than directly altering the stressor itself. Dysfunctional coping involves negative emotional responses used by caregivers to manage the stressor. A systematic review exploring coping strategies employed by caregivers of persons with dementia reported dysfunctional coping to be associated with higher distress levels and emotion‐focused coping to be linked with lower distress.^15^

However, existing studies looking at the mediating role of coping in the relationship between caregivers’ burden and distress^16^, ^17^ mostly examine each coping strategy in isolation,^16^, ^17^ even though caregivers typically employ multiple coping strategies simultaneously.^18^ Several qualitative studies show how caregivers employ multiple coping strategies.^19^, ^20^ For example, in response to PwSDs’ behaviors, caregivers may initially respond aggressively. They may eventually learn more effective responses to these behaviors or seek emotional support from friends and family. Caregivers often shift between these coping strategies in a short time frame. Collectively, these coping strategies impact caregivers’ outcomes. Focusing on one coping strategy in isolation without considering the impact of other coping strategies could potentially attribute the effect of one coping strategy to another. Hence, a comprehensive understanding of the effect of each coping strategy, while accounting for other coping strategies, is essential for tailoring interventions to support caregivers effectively during EOL conversations involving the communication of the terminal nature of dementia.

**RESEARCH IN CONTEXT**

1. **Systematic review**: The authors searched traditional sources to explore the relationship between caregivers’ understanding that dementia is terminal and their own distress. None has examined this for dementia caregivers. In addition, studies examine the relation of coping with distress but it is not clear if coping can mediate the above relationship.
2. **Interpretation**: Caregivers with a correct understanding of terminal illness had high distress levels but this was mediated through a greater use of dysfunctional and problem‐focused coping and lower use of emotion‐focused coping. Hence, it is how caregivers cope with the understanding that dementia is terminal that causes distress to caregivers.
3. **Future directions**: Caregivers’ understanding of terminal illness is important but it is associated with distress for caregivers. We show this is due to ineffective coping strategies, which helps to inform health care professionals about effective terminal illness disclosure. Future research should explore other strategies that might mediate or moderate this relationship.

Thus, in this article, we aim to assess the extent to which problem‐focused, emotion‐focused, and dysfunctional coping strategies simultaneously mediate the relationship between caregivers’ understanding of dementia as a terminal illness and their own distress.

**FIGURE 1:**
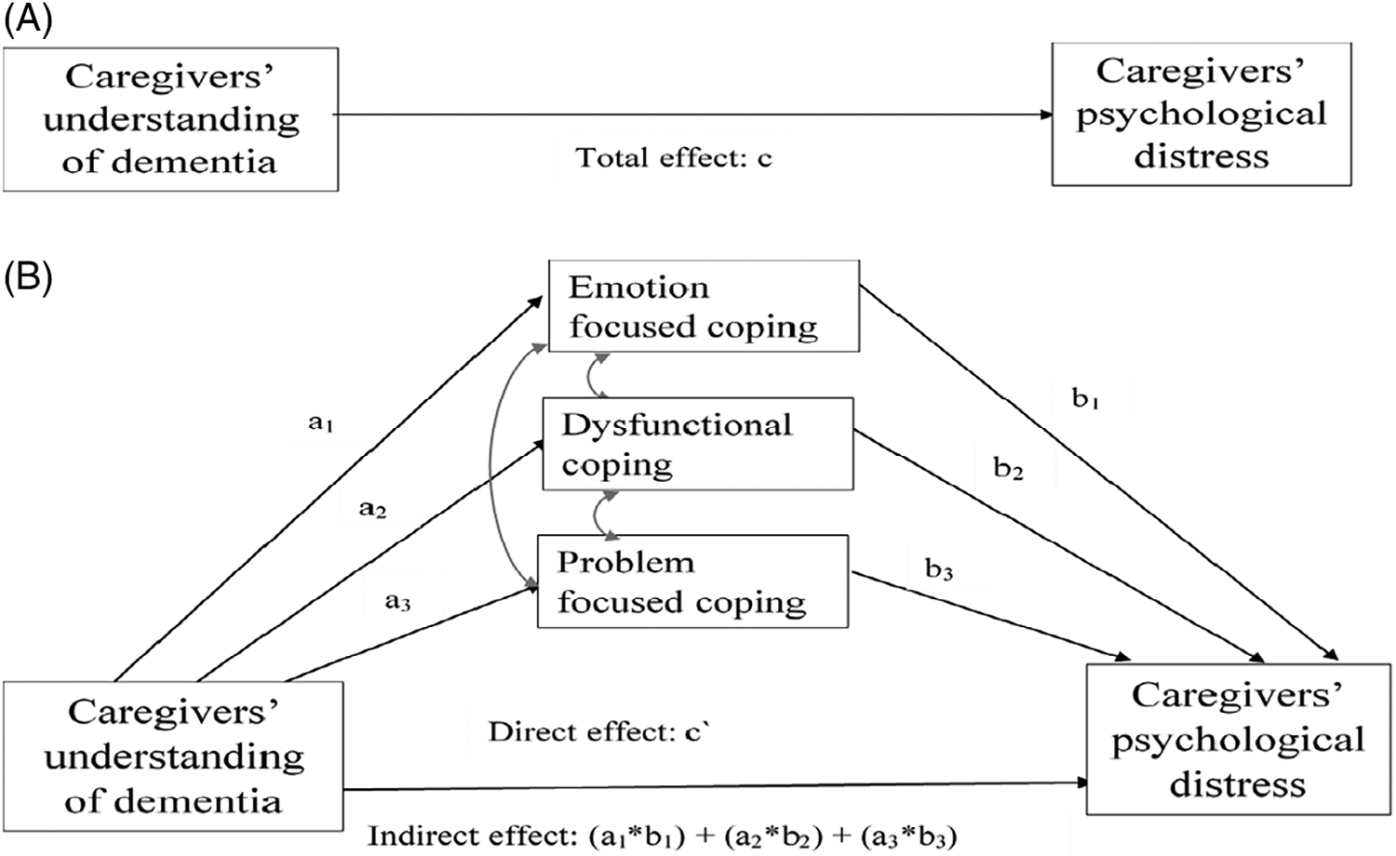
Path diagram illustrating the effect of caregivers’ terminal illness understanding and their distress.

The path diagram in Figure 1 summarizes our hypotheses as specified below:

H1: Caregivers who understand dementia as a terminal illness are more distressed, compared to caregivers who understand dementia is not terminal.

H2: Caregivers who understand dementia as a terminal illness use more of each coping strategy than caregivers who understand dementia is not terminal.

H3: Dysfunctional coping and problem‐focused coping are associated with higher distress. Emotion‐focused coping is associated with lower distress.

H4: Dysfunctional coping and problem‐focused coping mediate the positive relationship between caregivers’ understanding of dementia as a terminal illness and their distress, emotion‐focused coping partially offsets the relationship.

## 2 METHODS

### 2.1 Setting

The study was conducted in Singapore, a rapidly aging country in Southeast Asia. The prevalence of dementia is about 10% among older adults in the country.^21^ Consistent with Confucian values of filial piety, adult children often care for and make medical decisions for their older parents.^22^, ^23^

### 2.2 Study design and participants

We used data from the Panel study Investigating Status of Cognitively impaired Elderly in Singapore (PISCES), a prospective cohort of 215 primary informal caregivers of community‐dwelling PwSDs in Singapore. After obtaining written informed consent by caregivers, the study recruited caregivers of PwSDs (between May 2018 and March 2021) who met the inclusion criteria of having a diagnosis of dementia with Functional Assessment Staging Test stage 6C or higher (characterized by significant cognitive decline and help with daily activities [stage 6] or very severe decline characterized by losing the ability to communicate and requiring extensive care [stage 7]). Inclusion criteria for caregivers included being 21 years or older, being a Singapore citizen or permanent resident, being a family member and primary decisionmaker for the PwSD, meeting the PwSD at least once a week, and having intact cognition. We recruited eligible caregivers from memory/geriatrics clinics and general medicine wards of seven major public restructured hospitals in Singapore, six home care foundations, and two hospices. The study was approved by the appropriate institutional review board. Details of the study protocol have been published.^24^

### 2.3 Study variables

Caregivers were surveyed at baseline and every 4 months for a period of 3 years.

#### 2.3.1 Outcome variable

Caregiver distress was assessed using the Hospital Anxiety and Depression Scale (HADS),^25^ a composite measure of 14 items assessing caregiver feelings of anxiety and depression over the last week. Each item was scored from 0 to 3 and summed to create a total score of 0–42, with a higher score indicating higher distress.

#### 2.3.2 Independent variable

Caregivers’ understanding that dementia is a terminal disease: We asked caregivers to state their level of agreement with the statement “Dementia is a disease that you can die from.” Response options were based on a 6‐point Likert scale ranging from “completely agree” to “do not know.” Based on their response, we classified caregivers’ understanding of the terminal nature of dementia as correct (“Completely/partly agree”), unsure (“neither agree nor disagree/don't know”), or incorrect (“Completely disagree/partly disagree”).

#### 2.3.3 Mediating variables

Caregivers’ coping strategies (Table A1) were assessed using Brief‐COPE (Coping Orientation to Problems Experienced Inventory).^26^ We measured 13 coping strategies using two items for each strategy; each item scored from 1 to 4, with higher scores indicating greater use of that coping strategy (range: 1–8). We employed three subscales, as defined in the literature^27^: problem‐focused (including active coping, planning, and instrumental support), emotion‐focused (positive reframing, acceptance, humor, religion, and emotional support), and dysfunctional coping (including self‐distraction, denial, venting, behavioral disengagement, and self‐blame). The scores of these three subscales were normalized such that they ranged from 0 to 100.

#### 2.3.4 Control variables

We controlled for potential confounders. PwSDs’ health status was controlled using three variables: (1) *PWSDs’ quality of life* was assessed using 11 items from Quality of Life in Dementia (QUALID)^28^ and range from 1 to 55, with higher values signifying lower quality of life; (2) *PWSDs’ behavioral symptoms* were assessed using 14 items from the Cohen‐Mansfield Agitation Inventory^29^ and ranged from 14 to 70, with higher values indicating a greater extent of behavioral symptoms; and (3) *Functional impairment*: was assessed using seven items from the Bedford Alzheimer Nursing Severity‐Scale (BANS‐S)^30^ and ranged from 7 to 28, with higher values indicating greater impairment.

We also controlled for caregivers’ gender (1 = female), education (1 = above secondary), relationship with PwSD (1 = if caregiver is PwSD's child), relationship quality with PwSD (1 = quite/very close; 0 = not at all/little bit close), and caregiving burden. We assessed caregiving burden using 15 items from the modified Caregiver‐Reaction Assessment Scale^31^ and averaged to create a total score (range: 1–5). A higher score indicated a higher caregiving burden.

### 2.4 Statistical analysis

Our previous work has shown that caregivers’ understanding of dementia as a terminal illness changes over time, and not necessarily toward a correct perception.^32^ As caregivers’ understanding shifts in either direction, their coping and distress also change in response. We believe that these changes are concurrent rather than with a time lag of 4 months. Hence, we hypothesize that caregivers’ understanding of dementia as terminal affects their coping responses and hence their distress at the same time point. To test hypothesis 1, we estimated a mixed‐effects linear regression to assess the association between caregiver understanding of dementia as terminal (independent variable) and caregiver distress (outcome variable), controlling for the variables mentioned above.

To test hypotheses 2–4, we used a mediation model with multiple mediators. We estimated a generalized structural equation model with four equations. We included a random intercept in each equation at the level of caregiver (see A1 for details). Three equations estimated Path a (Figure 1)—the effect of caregivers’ understanding of dementia (categorical variable: correct, unsure [vs incorrect]) and caregivers’ coping (dysfunctional, emotion‐focused, and problem‐focused coping). The fourth equation estimated Path b (the effect of each coping strategy on caregivers’ distress) and Path c (the “direct effect” of caregivers’ perception of dementia on caregivers’ distress). The “indirect effect” was quantified as the product of Paths b and c. If the 95% confidence intervals (CIs) for the indirect effect did not include 0, mediation was considered significant.^33^ All analyses were conducted using STATA 17.0.

## 3 RESULTS

At baseline, we surveyed 215 caregivers of PwSDs. The PwSDs were on average 83.2 years old (SD: 8.1), mostly female (77.2%), Chinese (79.5%), and 30.7% had a spouse. Caregivers were, on average, 57.2 years of age (SD: 10.1), mostly female (69%), Chinese (79%), and the PwSD's spouse (13%)/child (75%) (Table 1).

**TABLE 1:** Baseline sample characteristics.

Caregiver characteristics	*N* (%)	Mean (SD)
Demographics		
Age		57.2 (10.1)
Female	149 (69.3)	
Chinese	170 (79.1)	
Education (above secondary)	119 (55.35)	
Relationship with person with severe dementia (PwSD)		
Spouse	28 (13.0)	
Adult child	161 (74.9)	
Others	26 (12.1)	
Psychological distress score, range: 0–35		9.9 (8.4)
Caregivers' understanding that dementia is terminal		
Correct	55 (25.6)	
Unsure	93 (43.3)	
Incorrect	67 (31.2)	
Coping strategies (normalized scores), range: 1–100		
Problem‐focused coping		61.9 (20.1)
Emotion‐focused coping		64.3 (15.8)
Dysfunctional coping		39.3 (12.4)
Caregiving burden, range: 1–5		2.9 (0.7)
Caregiver currently feels emotionally close to the patient	184 (85.6)	
PwSD characteristics		
Age		83.2 (8.1)
Female	166 (77.2)	
Chinese	171 (79.5)	
Married (has spouse)	66 (30.7)	
Quality of life score, range:11–54		23.1 (7.8)
Behavioral symptoms, range: 14–70		22.3 (8.2)
Functional impairment, range: 7–28		18.6 (4.3)

*N* = 215.

Abbreviations: PwSD, person with severe dementia; SD, standard deviation.

**Hypothesis 1**: At baseline, 43% of caregivers correctly understood dementia is a terminal illness, whereas 42% did not understand that dementia is a terminal illness. Caregivers’ mean distress scores varied significantly based on their understanding; it was highest for caregivers with a correct understanding, followed by caregivers who were unsure, and lowest for caregivers with an incorrect understanding (mean [SD]: 12.2 [9.4]; 10.4 [8.8]; 8.8 [7.6]). After controlling for confounders, caregivers who correctly understood dementia to be terminal and those who were unsure were more distressed compared to caregivers who believed that dementia was not terminal (correct: 𝛽 [95% CI]: 0.80 [0.00, 1.60]; unsure: 0.95 [0.04, 1.87]), thus supporting hypothesis 1 (Table 2).

**TABLE 2:** Relationship between caregivers’ terminal illness understanding and their psychological distress.

	Β	95% CI
Understanding of dementia (ref: incorrect)		
**Correct**	**0.80** **	**(0.00 to 1.60)**
**Unsure**	**0.95** **	**(0.04 to 1.87)**
PwSD's quality of life	0.16***	(0.10 to 0.22)
PwSD's behavioral symptoms	0.03	(–0.03 to 0.08)
PwSD's functional impairment	0.26***	(0.14 to 0.37)
Caregiving burden	0.89***	(0.56 to 1.22)
Caregiver is PwSD's child	1.76	(–0.54 to 4.06)
Caregiver gender (female)	−0.59	(−2.67 to 1.49)
Caregiver education (above secondary)	0.47	(−0.18 to 1.11)
Caregiver–PwSD relationship quality	−0.64	(−1.58 to 0.31)

Abbreviations: CI, confidence interval; PwSD, person with severe dementia.

** *p* < 0.05, ***p < 0.01.

**FIGURE 2 alz14102-fig-0002:**
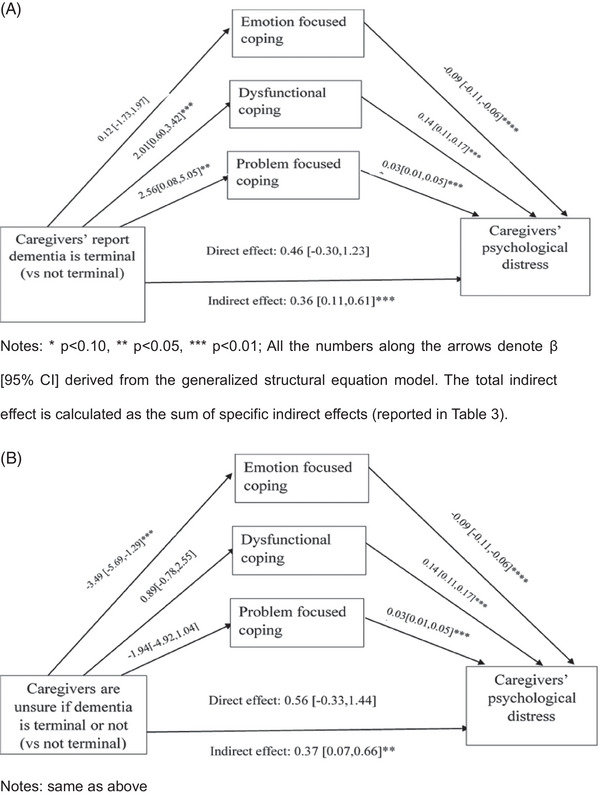
Results of the mediation analysis. (A) Caregivers’ distress when caregivers understand that dementia is a terminal illness (vs not a terminal illness). (B) Caregivers’ distress when caregivers are unsure about the terminal nature of dementia (vs not terminal).

**Hypothesis 2‐4**: The results from the multiple mediation model are summarized in Figure 2. Hypothesis 2 was partially supported. Caregivers who correctly understood dementia to be a terminal illness (vs those who were incorrect) employed more dysfunctional (𝛽 = 2.01; 95% CI 0.60 to 3.42) and problem‐focused coping strategies (𝛽 = 2.56; 95% CI 0.08 to 5.05). However, contrary to our hypothesis, these caregivers did not use more emotion‐focused coping. Of interest, caregivers who were unsure of the terminal nature of dementia used less emotion‐focused coping (𝛽 = ‐3.49; 95% CI ‐5.69 to ‐1.29), compared to caregivers with an incorrect understanding.

Hypothesis 3 was fully supported. Dysfunctional coping (𝛽 = 0.14; 95% CI 0.11 to 0.17) and problem‐focused coping (𝛽 = 0.03; 95% CI 0.01 to 0.05) were associated with higher distress and emotion‐focused coping (𝛽 = ‐0.09; 95% CI ‐0.11 to ‐0.06) was associated with lower distress.

Hypothesis 4 was partially supported. Although dysfunctional and problem‐focused coping mediated the positive association between caregivers’ understanding of the terminal nature of dementia and their distress, emotion‐focused coping did not offset this relationship. The specific indirect effect (IDE) (see Table 3) of caregivers’ correct understanding of the terminal nature of dementia on caregivers’ distress mediated through dysfunctional (IDE = 0.28; 95% CI 0.07 to 0.49) and problem‐focused coping (IDE = 0.09; 95% CI ‐0.01 to 0.19; *p* = 0.08) was significant. The total indirect effect of caregivers’ correct understanding of the terminal nature of dementia on caregivers’ distress mediated through all three coping strategies was also significant (IDE = 0.36; 95% CI 0.11 to 0.61). For caregivers who were unsure of the terminal nature of dementia, the specific indirect effect through emotion‐focused coping was significant (IDE = 0.31; 95% CI 0.09 to 0.53) and the total indirect effect (IDE = 0.37; 95% CI 0.07 to 0.66) mediated through all three coping strategies was significant.

**TABLE 3 alz14102-tbl-0003:** Specific indirect effects and direct effects of caregivers’ understanding of dementia as a terminal illness on caregivers’ distress.

	Direct effect on caregivers' distress		Specific indirect effect on caregivers' distress	
	Coeff	95% CI	Coeff	95% CI
Caregivers understand dementia is terminal (vs not terminal)	0.46	−0.30 to 1.23		
Dysfunctional coping			0.28	0.07 to 0.49
Emotion‐focused coping			−0.01	−0.17 to 0.15
Problem‐focused coping			0.09	−0.01 to 0.19
Caregivers are unsure if dementia is terminal or not (vs not terminal)	0.56	−0.33 to 1.44		
Dysfunctional coping			0.13	−0.11 to 0.36
Emotion‐focused coping			0.31	0.09 to 0.53
Problem‐focused coping			−0.07	−0.18 to 0.05

Abbreviation: CI, confidence interval.

## 4 DISCUSSION

This study investigates the relationship between caregivers’ understanding of the terminal nature of dementia, their coping strategies, and psychological distress. At baseline, only 43% of caregivers correctly understood dementia to be a terminal illness. Notably, caregivers with a correct understanding and those who were unsure experienced higher distress than those who understood incorrectly that dementia was not a terminal illness. Furthermore, dysfunctional and problem‐focused coping mediated the association between caregivers’ correct understanding and their distress, whereas emotion‐focused coping was a mediator in the association between caregivers’ “unsure” status of the terminal nature of dementia and their distress.

Many studies have investigated the relationship between patients’ terminal illness understanding and their psychological distress.^34^ However, less is understood about their caregivers’ terminal illness understanding and its impact on caregivers’ distress. Our finding that caregivers with “correct” understanding experienced higher distress compared to those with an “incorrect” understanding aligns with findings from a recent study involving caregivers of patients with advanced cancer. This study reported that even though caregivers’ correct terminal illness understanding benefitted patients, it was detrimental to caregivers’ psychological well‐being.^10^ Of interest, the finding that caregivers who were unsure about the terminal nature of dementia were also more distressed compared to those who were incorrect, suggests that uncertainty was not more favorable for caregivers. These results highlight the complexity of the relationship between caregivers' terminal illness understanding and their psychological well‐being.

Previous studies have highlighted the variability in coping strategies employed by caregivers, for instance, female caregivers tend to seek more social support and have wishful thinking compared to male caregivers.^35^, ^36^ However, little is known about how caregivers’ coping strategies may vary based on their terminal illness understanding. Our findings highlight that caregivers with a correct terminal illness understanding use more problem‐focused and dysfunctional coping strategies than caregivers with an incorrect terminal illness understanding. However, contrary to expectations, caregivers with correct terminal illness understanding did not employ more emotion‐focused coping strategies. Given that dementia is a terminal illness and there is limited control over its clinical course, caregivers who correctly understand it as terminal may be more cognizant of the terminal nature of dementia than caregivers with an incorrect understanding. The latter group may continue to harbor unrealistic expectations regarding life prolongation to preserve their hope. Prior studies have shown that lower perceived control over illness and treatment is associated with the use of more dysfunctional coping strategies and increased distress.^37^, ^38^ It is possible that the observed relationships between caregivers’ understanding of the terminal nature of dementia and their coping strategies reflect these underlying mechanisms. This highlights the importance of considering caregivers' perceptions of control in designing interventions to support their coping strategies.

We also found that[Fig alz14102-fig-0002] caregivers who are unsure about the terminal nature of dementia used significantly less emotion‐focused coping. Caregivers in this category may include those who are aware of the terminal nature of dementia but are unwilling or unable to admit it. This may impede their ability to employ emotion‐focused coping strategies, such as reframing or acceptance, since these strategies require acknowledging or accepting these difficult emotions.[Table alz14102-tbl-0003]

Consistent with previous evidence, we found that dysfunctional coping was associated with higher distress, and emotion‐focused coping was associated with lower distress.^15^ Although problem‐focused coping is shown to be associated with less distress in some studies,^39^ a meta‐analysis of studies involving caregivers of people with dementia shows no such association.^15^ In fact, evidence from longitudinal studies suggests a positive association between problem‐focused coping and distress,^27^ similar to what we found in this study. This may be explained by the fact that stressors in severe dementia, in this case, the fact that the condition is terminal, become less amenable to problem‐solving. The efficacy of the chosen coping strategy employed depends on the controllability of the stressor. According to Lazarus and Folkman,^12^ when the stressor itself is resistant to change, the most effective way to cope is to focus on reducing negative emotions resulting from the stressor rather than attempting to alter the stressor itself. In other words, an effective coping strategy entails using problem‐focused strategies when change is feasible and adapting emotionally when it is not. In the context of severe dementia, many problems that caregivers face are likely beyond their control, and hence coping emotionally rather than trying to mitigate the problem may be more effective, with the latter resulting in higher distress.^20^

Finally, the presence of significant mediation provides support for our conceptual model—caregivers with a correct understanding were more likely to use dysfunctional and problem‐focused coping strategies, thereby experiencing more distress than caregivers with an incorrect understanding. In contrast, caregivers who were unsure of the terminal nature of dementia were less likely to deploy emotion‐focused coping and through lower levels of this ‘protective’ coping strategy, experienced more distress than caregivers with an incorrect understanding. Our findings confirming our conceptual model extend previous research that has found associations between terminal illness understanding and distress^6^, ^7^, ^8^, ^9^, ^10^ (among patients with other terminal illnesses) and coping and distress^15^ but not tested the relationship between terminal illness understanding and coping or coping as a mediator. Findings suggest that it is *how* caregivers cope with the understanding that dementia is a terminal illness that causes distress to the caregiver. Previous studies among cancer patients show mixed evidence regarding the effect of prognostic awareness on the quality of life, anxiety, and depression of patients,^6^, ^7^, ^8^, ^9^ which raises the debate of whether or not prognostic communication is effective or even essential. Our findings suggest that understanding regarding the terminal nature of the disease will not increase the distress of caregivers if they use more emotion‐focused coping strategies than problem‐focused or dysfunctional coping strategies.

Our study has implications. First, we showed that caregivers who understood dementia as terminal as well as those who were unsure were more distressed. This suggests that without clear prognostic understanding, caregivers experience similar distress as when they are aware of the terminal condition but may be less‐prepared to make end‐of‐life decisions for the PwSD. Although caregivers who think dementia is not terminal are the least distressed, their incorrect understanding may translate to poor end‐of‐life care for PwSDs.^40^ Hence, caregivers must be provided with clear and complete prognostic information including what to expect along the clinical trajectory of dementia. Second, although caregivers with a correct terminal illness understanding had high distress levels, this seemed to be mediated through the greater use of dysfunctional and problem‐focused coping strategies and the lower use of emotion‐focused coping strategies. Hence, it appears that the source of distress is the way caregivers cope with the understanding that dementia is a terminal illness. Thus, providing terminal illness information along with interventions to support caregivers cope through the use of emotion‐focused coping can benefit both caregivers and PwSDs. For example, cognitive reframing interventions have been shown to reduce caregiver psychological morbidity.^41^ Similarly, caregiver support groups providing emotional support can relieve distress among caregivers.^42^ In the context of severe dementia, the use of problem‐focused coping may not be protective and may even be detrimental, as our results suggest, consistent with prior findings showing that interventions focusing on problem‐focused coping were not effective in reducing caregiver anxiety.^43^

The strength of our study is the use of a multiple mediator model to comprehensively assess the role of coping strategies in the relationship between caregivers’ awareness that dementia is terminal and their distress. Furthermore, our conceptual model was based on a theory, and validated instruments were used.

Our study is not without limitations. First, caregivers’ perception that dementia is terminal captures just one construct of their prognostic belief. Other constructs such as their caregivers' subjective life expectancy of PwSD should be explored in future studies. Second, although we used longitudinal data, the independent variable, outcome variable, and mediator variable were assessed at the same time point, as they all evolve simultaneously. Hence, we cannot infer causality. Third, there was attrition in our sample over time, due to the death of PwSDs and loss to follow‐up of their caregivers (8%–20% in each wave). Finally, our sample was not a probability sample, thereby limiting the generalizability of our results.

To conclude, we found that caregivers’ understanding of dementia as terminal was associated with their distress. However, mediation analysis showed that this relationship was fully mediated by the use of dysfunctional and problem‐focused coping strategies by caregivers, and that lower use of emotion‐focused coping did not offset this relationship. Hence, it is how caregivers cope with the understanding that dementia is a terminal illness that causes distress to the caregiver. Results suggest that terminal illness disclosure to caregivers should be accompanied by interventions to promote emotion‐focused coping strategies.

## CONFLICT OF INTEREST STATEMENT

The authors have no conflict of interest to disclose. Author disclosures are available in the supporting information.

## Supporting information



Supporting Information

Supporting Information
