# Supplementary material for: Caregiver coping mediates the relationship between caregivers’ understanding of dementia as terminal and their distress
Source: Alzheimers Dement. 2024 Jul 6;20(9):6606–14. doi: 10.1002/alz.14102 (PMC11497640; doi:10.1002/alz.14102)
Supplement: Supplementary file 1 — Supporting Information [file ALZ-20-6606-s002.docx]

**Table A1: Coping strategies in Brief-COPE**

| Problem focused coping  ***Active coping***  I've been concentrating my efforts on doing something about the situation I'm in.  I've been taking action to try to make the situation better.  ***Planning***  I've been trying to come up with a strategy about what to do.  I've been thinking hard about what steps to take.  ***Instrumental support***  I’ve been getting help and advice from other people.  I’ve been trying to get advice or help from other people about what to do. |
| --- |
| Emotion focused coping  ***Positive reframing***  I've been trying to see it in a different light, to make it seem more positive.  I've been looking for something good in what is happening.  ***Acceptance***  I've been accepting the reality of the fact that it has happened.  I've been learning to live with it.  ***Humor***  I've been making jokes about it.  I've been making fun of the situation (i.e. finding humour in the situation).  ***Religion***  I've been trying to find comfort in my religion or spiritual beliefs.  I've been praying or meditating.  ***Emotional support***  I've been getting emotional support from others.  I've been getting comfort and understanding from someone. |
| Dysfunctional coping  ***Self-distraction***  I've been turning to work or other activities to take my mind off things.  I've been doing something to think about it less, such as going to movies, daydreaming, shopping, sleeping etc  ***Denial***  I've been saying to myself "this isn't real."  I've been refusing to believe that it has happened.  ***Venting***  I've been saying things to let my unpleasant feelings escape.  I've been expressing my negative feelings.  ***Behavioral disengagement***  I've been giving up trying to deal with it.  I've been giving up the attempt to cope.  ***Self-blame***  I’ve been criticizing myself.  I’ve been blaming myself for things that happened. |

**Appendix 1: Mediation model with multiple mediators**

Mediation analysis is used to explain how an independent variable (X) affects a dependent variable (Y) through one or more potential intervening variables or mediators (M)(Baron and Kenny, 1986). In a simple mediation model as in Figure A1 (B), the causal effect of variable X on variable Y can be decomposed into an indirect effect via M and a direct effect (path c`). The indirect effect can be quantified as the product of the effect of X on M (path a) and the effect of M on Y (path b). The total effect of X on Y is given by path c (Figure 2A)(Baron and Kenny, 1986). The sum of the indirect effect and the direct effect should equal the total effect of X on Y .ie., c = c` + ab. This identity holds when M and Y are continuous(Preacher and Hayes, 2008).

*Figure A1: A simple mediation model*

**A**

**
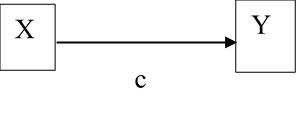
**

**B**

X

Y

c`

M

a

b

The hypothesized model in Figure 2 includes multiple mediators and is an extension to the simple mediation model(VanderWeele and Vansteelandt, 2014, Chen and Hsiang-Chin, 2016). As in a simple mediation model, Figure 1A represents the total effect of X on Y. Figure 1B represents the direct and indirect effects of X on Y. The *specific indirect effect* of X on Y via mediator *i* is the product of two unstandardized paths linking X to Y via M. For example, the specific indirect path of caregivers’ perception of dementia and their distress through dysfunctional coping is quantified as (a_2_ x b_2_). The *total indirect effect* of X on Y is the sum of specific indirect effects, in this case, (a_1_ x b_1_) + (a_2_ x b­_2_) + (a_3_ x b_3_­). The total effect is the sum of the direct and total indirect effect .i.e., c = c` + ((a_1_ x b_1_) + (a_2_ x b­_2_) + (a_3_ x b_3_­)).

Using a single multiple mediation model in lieu of multiple simple mediation models have several advantages. First, we can test the effect of any mediator, conditional on the presence of other mediators in the model. Second, it reduces omitted variable bias. Third, we can see if an overall effect of all mediators exist, when individual mediators affect Y in opposite directions. Lastly, we can assess the relative magnitude of the specific indirect effects allowing us to pit competing theories against one another(Preacher and Hayes, 2008).

In addition to using multiple mediators, since our data is longitudinal, we use a two-levelmediation model as follows(Hu et al., 2020).

$Y_{ij}= \beta_{0}+cX_{ij}+ \mu_{0j}+ \varepsilon_{ij}$ ------------------------------(1)

${M1}_{ij}= \beta_{0(1)}+a_{1}X_{ij}+ \mu_{0j(1)}+ \varepsilon_{ij(1)}$ ---------------------------(2a)

${M2}_{ij}= \beta_{0(2)}+a_{2}X_{ij}+ \mu_{0j(2)}+ \varepsilon_{ij(2)}$----------------------------(2b)

${M3}_{ij}= \beta_{0(3)}+a_{3}X_{ij}+ \mu_{0j(3)}+ \varepsilon_{ij(3)}$----------------------------(2c)

$Y_{ij}= \beta_{0(4)}+b_{1}{M1}_{ij}+b_{2}{M2}_{ij}+ b_{3}{M3}_{ij}{+ c`X_{ij}+\mu}_{0j(4)}+ \varepsilon_{ij(4)}$--------------------2(d)

In the above equations, *i* denotes each survey period, *j* denotes each caregiver.$Y$ is the continuous outcome variable (caregivers’ distress) and X is the categorical independent variable (caregivers’ perception of dementia). M1, M2 and M3 are the three mediator variables (emotion-focused, dysfunctional and problem-focused coping). Equation 1 is a mixed-effects model in which X is regressed on Y. The unstandardized coefficient c is the total effect. Equations 2(a)-2(d) are estimated simultaneously. In each equation, $\beta_{0}$ represents the fixed intercept, $\mu_{0j}$ represents the random intercept. We impose the following constraints – the covariance between the random intercepts in each equation is equal to zero and the three mediators are correlated (i.e., covariance between error terms of equations 2(a)-2(c) are non-zero).

**REFERENCES:**

BARON, R. M. & KENNY, D. A. 1986. The moderator-mediator variable distinction in social psychological research: conceptual, strategic, and statistical considerations. *J Pers Soc Psychol,* 51**,** 1173-82.

CHEN, L.-J. & HSIANG-CHIN, H. 2016. The indirect effect in multiple mediators model by structural equation modeling. *European Journal of Business and Economics,* 4**,** 36-43.

HU, X., ZHENG, J., FAN, T., SU, N., YANG, C. & LUO, L. 2020. Using Multilevel Mediation Model to Measure the Contribution of Beliefs to Judgments of Learning. *Front Psychol,* 11**,** 637.

PREACHER, K. J. & HAYES, A. F. 2008. Asymptotic and resampling strategies for assessing and comparing indirect effects in multiple mediator models. *Behavior Research Methods,* 40**,** 879-891.

VANDERWEELE, T. J. & VANSTEELANDT, S. 2014. Mediation Analysis with Multiple Mediators. *Epidemiol Methods,* 2**,** 95-115.
